# Re-stratification of patients with copy-number low endometrial cancer by clinicopathological characteristics

**DOI:** 10.1186/s12957-023-03229-w

**Published:** 2023-10-21

**Authors:** Li Liwei, Li He, Dai Yibo, Zhao Luyang, Shen Zhihui, Kang Nan, Shen Danhua, Wang Junzhu, Wang Zhiqi, Wang Jianliu

**Affiliations:** 1 Department of Obstetrics and Gynecology, Peking University People’s Hospital, Beijing, China; 2 The University of Texas MD Anderson Cancer Center UTHealth Houston Graduate School of Biomedical Sciences, The University of Texas MD Anderson Cancer Center, Houston, TX 77030 USA; 3 Department of Obstetrics and Gynecology, the Seventh Medical Center of Chinese, PLA General Hospital, Beijing, China; 4 Department of Pathology, Peking University People’s Hospital, Beijing, China; 5 The Big Data and Public Policy Laboratory, School of Government, Peking University, Beijing, China

**Keywords:** Endometrial cancer, Copy-number low, Molecular subtype, Clinicopathological characteristics, Recurrence, Nomogram

## Abstract

**Objective:**

To stratify patients with copy-number low (CNL) endometrial cancer (EC) by clinicopathological characteristics.

**Methods:**

EC patients who underwent surgery between June 2018 and June 2022 at Peking University People’s Hospital were included and further classified according to TCGA molecular subtyping: *POLE* ultramutated, microsatellite instability high (MSI-H), CNL, and copy-number high (CNH). Clinicopathological characteristics and prognosis of CNL patients were retrospectively reviewed. The Cox proportional hazards regression model was applied to perform univariate and multivariate analysis, and independent risk factors were identified. Differentially expressed genes (DEGs) according to overall survival (OS) were screened based on the transcriptome of CNL cases from the TCGA program. Finally, a nomogram was established, with an accuracy analysis performed.

**Results:**

(1) A total of 279 EC patients were included, of whom 168 (60.2%) were in the CNL group. A total of 21 patients had recurrence and 6 patients deceased, and no significant difference in recurrence-free survival (RFS) was exhibited among the four molecular subtypes (*P* = 0.104), but that in overall survival (OS) was statistically significant (*P* = 0.036). (2) CNL patients were divided into recurrence and non-recurrence groups, and significant differences (*P* < 0.05) were found between the two groups in terms of pathological subtype, FIGO stage, ER, PR, glycated hemoglobin (HbA1c), and high-density lipoprotein cholesterol (HDL-C). All the above factors were included in univariate and multivariate Cox regression models, among which pathological subtype, PR, and HDL-C were statistically different (*P* < 0.05), resulting in three independent risk factors for the prognosis of patients in the CNL group. (3) By comparing the transcriptome of tumor tissues between living and deceased CNL patients from the TCGA database, 903 (4.4%) DEGs were screened, with four lipid metabolism pathways significantly enriched. Finally, a nomogram was established, and internal cross-validation was performed, showing good discrimination accuracy with an AUC of 0.831 and a C-index of 0.748 (95% CI 0.444–1.052). (4) According to the established nomogram and the median total score (85.89), patients were divided into the high score group (*n* = 85) and low score group (*n* = 83), and the 8 patients with recurrence were all in the high score group. Survival analysis was performed between the two groups, and the difference in RFS was statistically significant (*P* = 0.010).

**Conclusion:**

In the CNL group of EC patients, pathological subtype, PR, and HDL-C were independent prognostic risk factors, the nomogram established based upon which had a good predictive ability for the recurrence risk of patients with CNL EC.

**Supplementary Information:**

The online version contains supplementary material available at 10.1186/s12957-023-03229-w.

## Introduction

Endometrial cancer (EC) is one of the three major cancers of the female reproductive system, with an incidence rate ranking 4th among all cancers and showing high heterogeneity in histological, genetic, and molecular characteristics [1]. In 2013, the Cancer Genome Atlas (TCGA) Project [2] applied a multi-omic analysis and re-classified EC into four molecular subtypes, including *POLE* ultramutated, microsatellite instability-high (MSI-H), copy-number low (CNL), and copy-number high (CNH), which have started a new era of diagnosis and treatment by combining histopathological characteristics and molecular subtyping [2]. However, the large proportion of patients in the CNL group [3] (especially in the Chinese population [4], the absence of specific molecular patterns, and the large difference in prognosis, greatly require further refinement of stratification to guide the management of CNL patients. In this study, we presented a retrospective analysis of EC patients who underwent TCGA molecular subtyping in our institution in recent years to explore the feasibility of re-stratification for CNL patients based on clinicopathological characteristics.

## Materials and methods

### Study subjects and data collection

1. Study subjects: a total of 279 patients with EC who underwent surgical treatment and molecular subtyping between June 2018 and June 2022 at Peking University People’s Hospital were collected. Patients’ clinicopathological data including age, body mass index (BMI), menopausal status, metabolic syndrome-related comorbidities, comorbid malignancies of other organs, family history of cancer, preoperative serum CA125 and human epididymis protein 4 (HE4) levels, stage, pathological subtype, tumor grade, myometrial invasion, lymphovascular space invasion (LVSI), lymph node metastasis, tumor maximum diameter, and immunohistochemical parameters (including ER, PR, etc.) et al. were collected from the medical record system. All pathological reviews were finished in the Department of Pathology of Peking University People’s Hospital by two independent gynecologic pathologists. The staging was determined according to the International Federation of Gynecology and Obstetrics (FIGO) 2009 staging system [5]. Histopathological classification was performed according to the World Health Organization (WHO) 2014 classification system. The grading of tumors was in accordance with the FIGO criteria [6]. This study was approved by the biomedical ethics committee of Peking University People's Hospital, with informed consent from all participants. To explore differentially expressed genes (DEGs) associated with overall survival (OS) in CNL patients, we also extracted mRNA expression profiles from the Uterine Corpus Endometrial Carcinoma (TCGA, PanCancer Atlas) database through cBioPortal [7, 8].
2. We tried to control selection bias by expanding the sample size as much as possible and strictly screening the inclusion and exclusion criteria. Inclusion criteria: The pathological diagnosis was endometrial cancer; There was no abnormal liver and kidney function and bone marrow suppression before the operation. The subjects voluntarily joined the study, signed the informed consent, had good compliance, and cooperated with the follow-up. Exclusion criteria: previous history of other malignancies or concurrent malignancies; Unwilling to accept follow-up.
3. Follow-up: patients were followed up every 3 months for 2 years after completing treatment and every 6 months for 3 years thereafter. The follow-up contents mainly included imaging (including pelvic abdominal ultrasound or CT) examination and serum tumor marker. The last follow-up date was December 31, 2022, and 279 patients were followed up for a median of 16.9 months (range 0.7–70.7 months) postoperatively, with the rate of follow-up being 100%.

### Molecular subtyping and immunohistochemical detection

1. Molecular subtyping: endometrial cancer tissue and blood samples from patients were collected, sequencing was performed using a high-throughput sequencer (Illumina products, USA), and sequencing data were analyzed using a high-throughput sequencing data analysis system (Amoy Diagnostics Co., Ltd.). Based on the sequencing results, in this study, we refer to the 2013 TCGA molecular classification method [2] to classify the patients into four subtypes: *POLE* ultramutated, MSI-H, CNL, and CNH, and the specific subtyping was performed using Trans-PORTEC [9], as follows: (1) the mutation status of the *POLE* gene was detected if the pathogenic mutation of the *POLE* gene was judged to be *POLE* ultramutated; (2) a microsatellite instability (MSI) value ≥ 0.4 in a sample wild-type for the *POLE* gene was judged as MSI-H type; (3) *TP53* mutation status was determined in microsatellite stable patients and CNH and cases without mutation were judged as CNL [10].
2. Immunohistochemistry: ER, PR, p53, and Ki-67 expression were detected by immunohistochemistry with an envision kit purchased from Zymed (USA).

### Statistical analysis

SPSS 25.0 software was used for statistical analysis. Quantitative data were tested for normality and those with a normal distribution were given $\overline{x }$±s indicated, by independent samples t-test or one-way ANOVA; Measures that are not normally distributed are presented as the median (25th–75th percentile) [M(P25–p75)] with the nonparametric Mann‑Whitney *U* test or Kruskal‑Wallis *H* rank sum test. The counting data were tested for the *χ*^2^-test or Fisher’s exact probability method. *P* < 0.05 was taken as statistically significant. Univariate and multivariate analyses were performed using Cox proportional hazards regression models to identify factors associated with prognosis in the CNL group; These factors were determined with hazard ratios (HR) and 95% confidence intervals (CI). As for the TCGA database, DEGs of tumor tissues between living and deceased patients were screened by the ‘limma’ package and focused on whether DEG was related to glucose, sex hormones, or lipid metabolism pathways [11].

The final model selection for the nomogram was performed by a backward step-down selection process using a threshold of *P* < 0.05, and factors without clinical significance were also excluded from the model. ROC curves were used to find the cut-off value and evaluate the discriminatory ability of the model. All statistical analyses were 2-tailed and *P* < 0 0.05 were deemed statistically significant. The DEG analysis, nomogram, and time-dependent ROC were established with R (http://www.R-project.org) and EmpowerStats software (www.empowerstats.com, X&Y Solutions, Inc. Boston, MA, USA).

## Results

### Clinicopathological and molecular features of EC cases

**Fig. 1:**
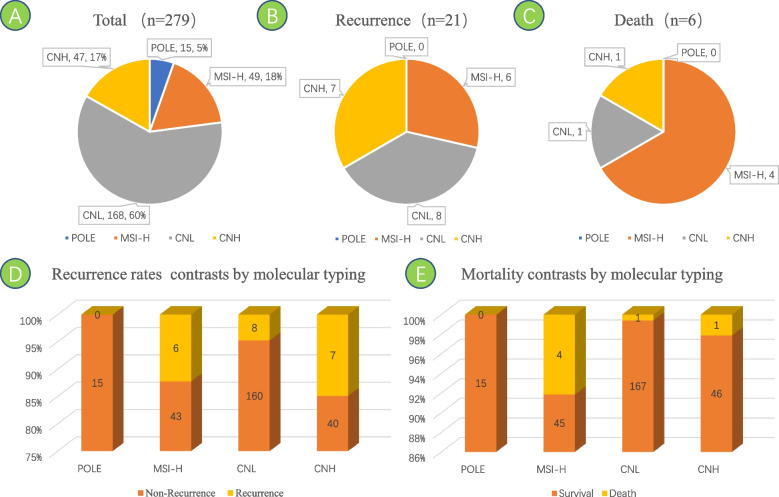
The proportion of four molecular subtypes in the total EC cohort, the number of patients with recurrence and death. The recurrence rates and mortality contrasts by molecular subtyping

A total of 279 patients were included, of whom 15 (5.4%) were in the *POLE* ultramutated group, 49 (17.6%) in MSI-H, 168 (60.2%) in CNL, and 47 (16.8%) in CNH (Fig. 1A).

Postoperative follow-up as of December 31, 2022, a total of 21 patients had recurrence, including 0 in the *POLE* ultramutated group, 6 in MSI-H (12.2%), 8 in CNL (4.8%), and 7 in CNH (14.9%) (Fig. 1B, D). A total of 6 patients died, including 0 in the *POLE* ultramutated group, 4 in MSI-H (8.2%), 1 in CNL (0.6%), and 1 in CNH (2.1%) (Fig. 1C, E).

**Fig. 2:**
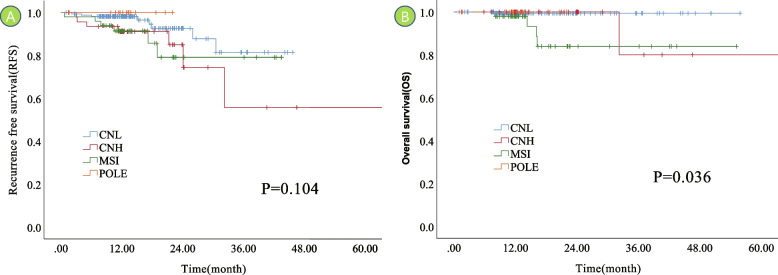
Kaplan–Meier analysis for overall survival (OS) and recurrence-free survival (RFS) in EC patients stratified by molecular subtyping

The survival analysis showed that the 1-year recurrence-free survival rate (RFS) of patients in *POLE* ultramutated group was 100%, and the 3-year RFS of patients in MSI‑H, CNL, and CNH groups were 79.1%, 81.2%, and 55.8%, respectively, with no statistically significant difference (*P* = 0.104) (Fig. 2A); the 1-year OS of patients in the *POLE* ultramutated group was 100%, and the 3-year OS of patients in MSI‑H, CNL, and CNH groups were 84.0%, 99.4%, and 80.0%, respectively. The difference was statistically significant (*P* = 0.036) (Fig. 2B).

Because of the large proportion of patients and the large difference in prognosis in the CNL group, they were further analyzed. Clinicopathological data of patients in the recurrence (*n* = 8) and non-recurrence (*n* = 160) groups are shown in Table 1. There were 8 non-endometrioid carcinoma patients: 2 patients in the recurrence group: 1 serous carcinoma and 1 undifferentiated carcinoma; 6 patients in the non-recurrence group: 2 serous carcinomas, 1 mixed carcinoma, 1 clear cell carcinoma, 1 carcinosarcoma, 1 mucinous carcinoma. There was no statistical difference between the two groups in surgical approach and postoperative adjuvant radiotherapy, chemotherapy, and endocrine therapy, but significant differences were exhibited in pathological subtype, FIGO stage, ER, PR, HbA1c, and HDL-C (Table 1).

**Table 1:** Baseline characteristics of the CNL subgroup

Variables	Total	Recurrence(*n* = 8)	Non-recurrence(*n* = 160)	*P* value
Clinical and pathological				
Age (years)($\overline{x }$±s)	56.11 ± 8.93	58.75 ± 11.67	55.97 ± 8.80	0.393
BMI ((kg/m^2^)($\overline{x }$±s)	26.58 ± 3.73	26.94 ± 2.05	26.56 ± 3.80	0.782
Pathological type (*n*)(%)				0.035
Endometrioid adenocarcinoma	159	5(71.4)	154(96.3)	
Non-endometrioid adenocarcinoma	8	2(28.6)	6(3.8)	
Myometrial invasion (*n*)(%)				0.360
< 1/2	127	4(57.1)	123(76.9)	
≥ 1/2	40	3(42.9)	37(23.1)	
FIGO (*n*)(%)				0.038
Early (stage I–II)	147	4(57.1)	143(89.4)	
Advanced (stage III–IV)	20	3(42.9)	17(10.6)	
Grade (*n*)(%)				0.062
Low (Grade 1–2)	153	4(66.7)	149(93.7)	
High (Grade 3)	12	2(33.3)	10(6.3)	
LVSI (*n*)(%)				0.159
Negative	129	4(57.1)	125(80.1)	
Positive	34	3(42.9)	31(19.9)	
Cervical invasion (*n*)(%)				0.616
No	152	6(85.7)	146(91.3)	
Yes	15	1(14.3)	14(8.8)	
Lymph node metastasis (*n*)(%)				0.052
No	155	4(66.7)	151(94.4)	
Yes	11	2(33.3)	9(5.6)	
Immunohistochemistry				
ER(%)[M(P25,P75)]	70[50,90]	10[0,72.5]	70[50,90]	0.015
PR(%)[M(P25,P75)]	75[50,90]	15[0,50]	80[50,90]	0.005
Ki-67(%)[M(P25,P75)]	30[20,40]	40[22.5,67.5]	30[20,40]	0.158
Metabolic indexes				
FBG(mmol/L)[M(P25,P75)]	5.8[5.2,6.8]	5.9[5.2,9.2]	5.8[5.2,6.8]	0.582
FINS(uU/ml)[M(P25,P75)]	12.9[7.7,12.9]	12.9[10.6,13.8]	12.9[7.4,12.9]	0.341
HbA1c(%)[M(P25,P75)]	6.8[6.8,6.8]	7.3[6.8,8.0]	6.8[6.8,6.8]	0.002
TC(mmol/L)[M(P25,P75)]	5.0[4.3,5.8]	4.6[3.8,6.2]	5.0[4.3,5.8]	0.546
TG(mmol/L)[M(P25,P75)]	1.6[1.2,2.1]	1.5[1.2,2.9]	1.6[1.2,2.1]	0.955
HDL-C(mmol/L)[M(P25,P75)]	1.2[1.0,1.4]	0.9[0.8,1.2]	1.2[1.0,1.4]	0.035
LDL-C(mmol/L)[M(P25,P75)]	3.1[2.6,3.7]	3.2[2.3,4.1]	3.1[2.6,3.7]	0.955
HOMAIR[M(P25,P75)]	3.1[2.2,4.1]	4.0[3.0,5.8]	3.1[2.1,4.1]	0.146
Testosterone(nmol/L)[M(P25,P75)]	1.3[1.1,1.5]	1.3[1.3,1.3]	1.3[1.1,1.5]	0.912
Treatment				
Surgical approach (*n*)(%)				0.079
Open	73	6(75.0)	67(41.9)	
Minimally invasive	95	2(25.0)	93(58.1)	
Postoperative chemotherapy (*n*)(%)				0.132
No	108	3(37.5)	105(66.0)	
Yes	59	5(62.5)	54(34.0)	
Postoperative radiotherapy (*n*)(%)				0.438
No	117	7(87.5)	110(69.2)	
Yes	50	1(12.5)	49(30.8)	
Postoperative endocrine therapy (*n*)(%)				
No	143	5(83.3)	138(86.3)	1.000
Yes	23	1(16.7)	22(13.8)	

*BMI* body mass index, *LVSI* lymph‑vascular space invasion, *ER* estrogen receptor, *PR* progesterone receptor, *FBG* fasting blood glucose, *FINS* fasting insulin, *TC* total cholesterol, *TG* triglyceride, *HDL-C* high-density lipoprotein cholesterol, *LDL-C* low-density lipoprotein cholesterol, *HOMAIR* homeostasis model assessment of insulin resistance

### Predictors for survival in the CNL group

**Fig. 3:**
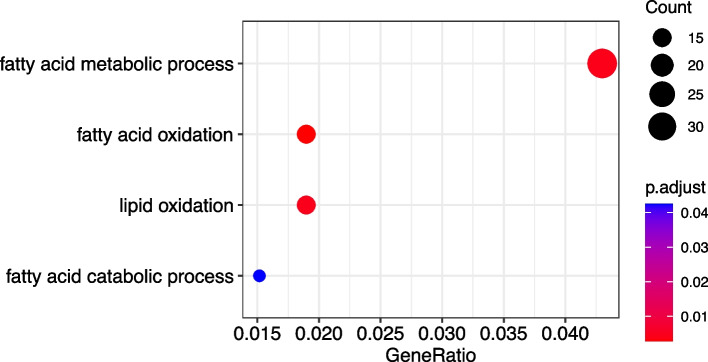
Various pathways associated with lipid metabolism were enriched when comparing transcriptome of tumor tissue from living and deceased CNL patients

The pathological subtype, FIGO stage, ER, PR, HbA1c, and HDL-C were included in the univariate and multivariate Cox regression model analysis (Table 2), in the multivariate Cox analysis, the pathological subtype (HR 0.053, 95% CI 0.008–0.363), PR (HR 0.969, CI 0.945–0.994), and HDL-C (HR 0.059, CI 0.005–0.646) were statistically different, thus the three factors were independent risk factors for prognosis in the CNL group. Further transcriptomic analysis of the TCGA database screened 903 (4.4%) DEGs between living and deceased CNL patients (Supplementary Table S1 and Supplementary Figure S1), Gene Ontology (GO) analysis based on which enriched 141 pathways in the biological process (BP) group (Supplementary Table 2), with 4 pathways associated with lipid metabolism (Fig. 3).

**Table 2:** Results of multivariate Cox regression analysis

Variables	HR	95%CI	*P* value
Clinical and pathological			
Pathological type	0.053	0.008–0.363	0.003
FIGO	0.459	0.075–2.802	0.399
Immunohistochemistry			
ER	1.001	0.965–1.038	0.961
PR	0.969	0.945–0.994	0.017
Metabolic indexes			
HbA1c	1.305	0.601–2.835	0.501
HDL-C	0.059	0.005–0.646	0.020

*ER* estrogen receptor, *PR* progesterone receptor, *HDL-C* high-density lipoprotein cholesterol

### Construction of a nomogram for the prediction of recurrence in the CNL group

**Fig. 4:**
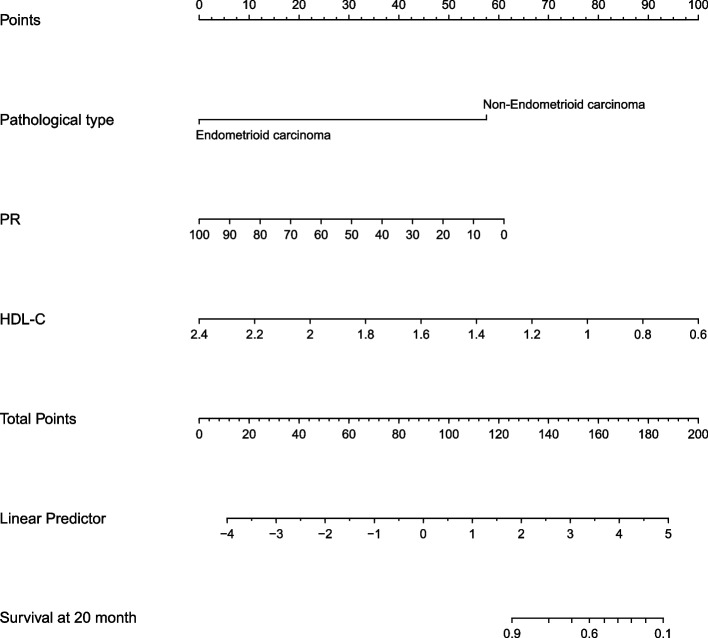
Nomogram predicting the recurrence of CNL group

Based on results from the multivariate Cox regression model, a nomogram was constructed and incorporated clinical variables, including pathological subtype, PR, and HDL-C. For individualized prediction, draw an upward vertical line to the “points” bar to calculate the total points corresponding to the patient’s characteristics. Then, draw a downward vertical line from the “total points” line based on the sum to calculate the risk of recurrence 20 months after surgery (Fig. 4).

### Accuracy of the nomogram

**Fig. 5:**
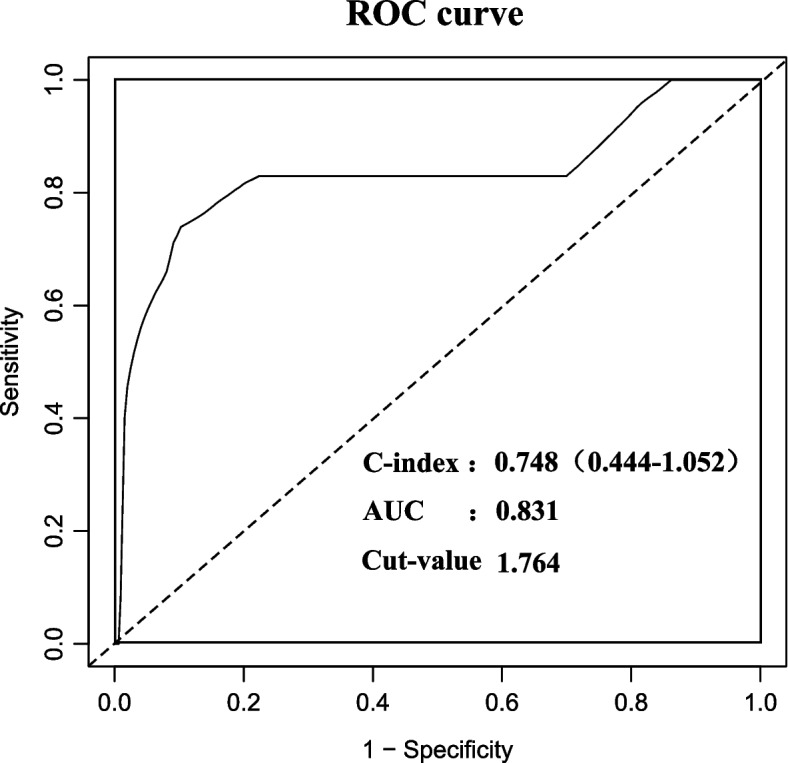
Receiver operating characteristic curves of internal verification corresponding nomogram to predict the recurrence of CNL group

The nomogram was cross-validated internally by the 500 repetitions of bootstrap sample corrections. For the prediction of the recurrence of the CNL group, the nomogram showed good discrimination accuracy with an AUC of 0.831 and a C-index of 0.748 (95% CI 0.444–1.052) in internal validation (Fig. 5).

**Fig. 6:**
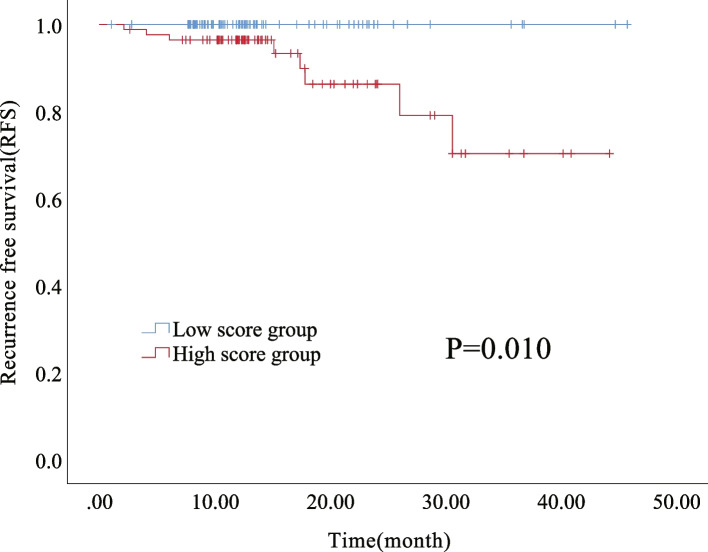
Kaplan–Meier analysis for RFS in CNL group with low score group and high score group

Based on the established nomogram, the median total score of each CNL patient (*n* = 168) was calculated to be 85.89, and patients were divided into low (total score < 85.89, *n* = 83) and high (total score ≥ 85.89, *n* = 85) score groups according to which. All 8 patients with recurrence were in the high-score group. Survival analysis was performed and the difference was statistically significant (*P* = 0.010) (Fig. 6).

## Discussion

Since 2013, TCGA-based molecular subtyping has provided an important reference value for judging the prognosis and treatment of EC patients, shows good application prospects, and has been included in the National Comprehensive Cancer Network (NCCN) guidelines [12] to recommend clinical application. In this study, the enrolled EC patients were molecularly typed according to TCGA criteria [2] by detecting *POLE* mutation, microsatellite stability status, and TP53 mutation status using high-throughput sequencing technology, the clinicopathological characteristics of EC patients were retrospectively analyzed, and prognosis analysis was performed, because of the large proportion of patients in the CNL group and the great difference in prognosis, further analysis was performed to identify 3 independent risk factors for prognosis. Nomograms [13–15], which is an intuitive and easily readable graphical chart based on the results of the logistic regression or Cox regression, could accurately predict the probability of occurrence of an event. For clinical application, the nomogram could incorporate patient individual characteristics and need further validation by cross-validation and bootstrapping methods. In the current study, we constructed a nomogram based on three clinicopathological parameters to predict the risk of recurrence for patients in the CNL group. The exploration of re-stratification of patients in the CNL group by clinicopathological characteristics was initially explored.

### Endometrial cancer molecular subtyping and clinicopathological characteristics

In 2013, TCGA project [2] performed an integrated genomic, transcriptomic, and proteomic study of 373 endometrial cancer patients, including 307 endometrioid, 53 serous, and 13 mixed carcinomas, and classified endometrial cancers into four molecular subtypes: *POLE* ultramutated (7%), MSI‒H (28%), CNL (39%), and CNH (26%), Among them, *POLE* ultramutated type had the best prognosis [16], MSI‒H patients and CNL patients had the intermediate prognosis, while CNH patients had the worst prognosis. In 2020, TCGA molecular typing assays were first incorporated into the National Comprehensive Cancer Network (NCCN) guidelines; In the same year, molecular typing of endometrial cancer was included in the 5th edition of the WHO classification of tumors of the female reproductive organs. Molecular subtyping of endometrial cancer is becoming more widely used.

Molecular subtyping was different and also brought about differences in clinicopathological and immunomolecular features, and the study showed that the stage, pathological type, and grade of the four molecular subtypes patients were all statistically different, with the *POLE* ultramutated, MSI-H, and CNL subtypes more prone to early clinical stage (higher proportion in FIGO stage I–II) and endometrioid adenocarcinoma, while the CNH type patients were more III-IV and non-endometrioid adenocarcinoma at diagnosis. In endometrioid adenocarcinoma, the *POLE* ultramutated and CNL subtypes are mainly highly differentiated. The MSI and CNH subtypes are mostly low and medium differentiated. The positive expression rate of PD-L1 in patients with the MSI-H subtype was significantly higher than that in patients with *POLE* ultramutated, CNL, and CNH subtypes.

However, currently used molecular typing still has certain limitations. A large proportion of endometrial cancer patients are classified as CNL type [17], limiting the use of molecular subtyping for prognosis determination and treatment selection in these patients.

Thus, in recent years, numerous studies have been developed to refine the clinical management and personalization of patient therapy with EC, considering not only traditional prognostic factors but also an innovative molecular analysis with the aim of defining different classes of risk and developing therapies targeted to the molecules involved in carcinogenesis [18].

The 2021 joint guidelines of the European Society of Gynecological Oncology, European Society for Radiotherapy and Oncology, and European Society of Pathology (ESGO/ESTRO/ESP) for the management of patients with EC encourage molecular classification, especially in high-grade tumors, and propose a new prognostic risk stratification based on both histological and molecular features [19]. However, how the molecular signature can be integrated with classic pathological factors is still under investigation.

Recent studies [20] have shown that LVSI has a prognostic value independent of TCGA signature, as well as age and adjuvant treatment, increasing the risk of death of any cause, death due to EC, and recurrent or progressive disease by 1.5–2 times. Deep myometrial invasion has been shown to affect the risk of recurrence independently from the TCGA groups, but not the risk of death of any cause [21]. In addition, there are other histopathological features, not considered in the current guidelines, that were proposed as possible independent prognostic factors, such as microcystic, elongated, and fragmented (MELF) patterns of invasion and tumor budding [22–24]. The prognostic significance of these factors, their reproducibility, and their possible integration into the current risk stratification system require further investigation.

### Endometrial cancer and metabolic syndrome

Studies [25, 26] have shown that obesity and metabolic abnormal diseases such as hyperlipidemia, hypertension, diabetes, and hyperinsulinemia are associated with endometrial cancer incidence, adverse pathological features, and poor prognosis. Epidemiological studies have shown that overweight patients (BMI ≥ 25 kg/m^2^) have a 2.45-fold higher risk of developing EC and diabetic patients a 2.12-fold higher risk [27]. In addition, obesity-associated insulin resistance, sedentary lifestyle, Lynch syndrome, nulliparity, early menarche, and anovulation are potential risk factors for EC. Among them, insulin and insulin-like growth factor-1 (IGF-1) promote EC cell proliferation and migration through PI3K/Akt and RAS/MAPK pathways [28].

Another study from our unit [26] showed that Metabolic syndrome (Mets) was strongly associated with advanced-stage, high-grade, positive lymph node metastasis, LVSI positivity, and deep myometrial invasion in endometrial cancer patients, in which HDL-C was an independent risk factor for EC. To further evaluate the ability of HDL-C to predict the prognosis of EC patients, ROC analysis was performed, and the areas under the curve (AUC) at 1, 3, and 5 years were 0.626, 0.599, and 0.648, respectively. Based on HDL-C, grade, and stage, nomograms were constructed to predict the 1- and 3-year survival rates of EC patients. And the prediction performance is good.

Therefore, several metabolic-related indexes including FBG, FINS, HbA1c, TC, TG, HDL-C, and LDL-C were included in this study to conclude that HDL-C is an independent risk factor for the prognosis of patients in the CNL group. For the re-stratification of CNL patients at the molecular level, we also designed a transcriptomic analysis based on tumor tissues from the TCGA database. We found 903 DEGs between EC from living and deceased CNL patients (Supplementary Table S1 and Supplementary Figure S1), and 4 lipid metabolism pathways were enriched (Fig. 4), suggesting the role of lipid metabolism in the progress of CNL EC and the rationality of incorporating molecular features into the re-stratification of CNL EC. However, no glucose or sex hormone metabolism pathways were enriched, probably due to the relatively small number of total CNL patients (*n* = 149) and those with recurrence (*n* = 12) in the TCGA database. However, given the relatively large proportion of CNL patients in EC patients from China [4] and the wide application of second-generation sequencing (or even single-cell RNA sequencing) in scientific research [29], it is believed that a practical re-stratification strategy combining clinicopathological and molecular in CNL patients will soon be proposed.

### The endometrial cancer immune microenvironment

In endometrial cancer, as in many cancers, the immune microenvironment plays an important role in cancer progression and therapeutic response. This includes both tumor-stroma interactions and tumor-infiltrating immune cell interactions. The tumor immune microenvironment (TIME) is composed of immune cells, mesenchymal cells, endothelial cells, inflammatory mediators, and extracellular matrix molecules [30]. The occurrence and development of EC are closely related to the regulation of the TIME. Through a series of mechanisms, tumor cells eventually escape the surveillance of the immune system and inhibit the cytotoxic effects of immune cells [31].

A series of recent studies have immunophenotyped EC according to immune-related genes or immune cell infiltration. Included: Li and Wan [32] defined four immunophenotypes, C1(immunodepression), C2(IFN‐ɣ dominant type), C3(inflammatory type), and C4(immunologically balanced type), using immune-related gene signatures from the TCGA database combined with gene-set variant analysis and hierarchical clustering. Cai et al. [33] clustered the samples according to the infiltration of immune cells in tumor tissues and obtained three subpopulations with high, intermediate, and low immune cell infiltration, namely, C I, C II, C III, among which C I and C II patients performed adjuvant treatment better. Another study from our group [10], combining molecular subtyping with the immune microenvironment, explored the immune microenvironment characteristics of different molecular subtyping, in which the tumor mutation burden (TMB) levels of *POLE* mutant and MSI-H cases were significantly higher than that of the other two subtypes (*P* < 0.001). He combined *POLE* mutant and MSI-H subtypes into the TMB high (TMB-H) subtype. The TMB-H subtype showed a high degree of infiltration of CD8 + T cells. Concluded that EC of TMB-H, no specific molecular profile (NSMP), and TP53 mutant subtypes displayed phenotypes of the normal immune response, absence of immune infiltration, and suppressed immune response, respectively. These features may provide mechanistic explanations for the differences in patients’ prognosis and efficacy of immune checkpoint blockade therapies among different endometrial cancer subtypes.

These studies all illustrate that the immune microenvironment and immunophenotyping may be the next areas we should focus on, and combining molecular subtyping and immune signature may be more helpful in guiding the prognosis of patients and selecting the patients who are suitable for immunotherapy, which will facilitate a more individualized diagnosis and treatment of patients. Therefore, our group will further incorporate tumor tissue immune microenvironment indicators in the future to make immune scores for patients, strive to combine multi-dimensional indicators, establish an early warning model of endometrial cancer prognosis, and implement a precision stratified diagnosis and treatment for patients. Limitation of this study: on the one hand, this study was retrospective and included insufficient cases, some missing data, insufficient follow-up time, and a low number of patients with recurrence and death, leading to possible bias in the results. The established model lacks external validation and awaits further validation. On the other hand, this study only explored the effect of clinical indicators on prognosis and did not explore the deep mechanism, which was the focus of our next research.

## Conclusion

In this study, further stratification of endometrial cancer patients with CNL type was explored and attempted, three independent risk factors including pathological type, PR, and HDL-C were sought, based on which a nomogram was constructed and validated for accuracy. This model can be quite instructive for the prognosis of patients.

### Supplementary Information

**Additional file 1: Supplementary Figure S1.** DEGs between living and deceased CNL patients from TCGA database.

**Additional file 2: Supplementary Table S1.** DEGs between living and deceased CNL patients from TCGA database.

**Additional file 3: Supplementary Table S2.** Gene Ontology (GO) analysis.

## Data Availability

All data of this research are from Peking University People's Hospital databases, and algorithms and annotated data in this research can be requested from the corresponding author based on academic collaboration.

## Abbreviations

EC: Endometrial cancer
POLE: Polymerase epsilon
MSI-H: High microsatellite instability
CNH: Copy number high
CNL: Copy number low
RFS: Recurrence-free survival
OS: Overall survival
BMI: Body mass index
LVSI: Lymph‑vascular space invasion
ER: Estrogen receptor
PR: Progesterone receptor
FBG: Fasting blood glucose
FINS: Fasting insulin
TC: Total cholesterol
TG: Triglyceride
HDL-C: High-density lipoprotein cholesterol
LDL-C: Low-density lipoprotein cholesterol
HOMA-IR: Homeostasis model assessment of insulin resistance
NCCN: The National Comprehensive Cancer Network
HR: Hazard ratio
CI: Confidence interval
ROC: Receiver operating characteristic
AUC: Area under the curve
C-index: Concordance Index
